# Ser276 Phosphorylation of NF-kB p65 by MSK1 Controls SCF Expression in Inflammation

**DOI:** 10.1371/journal.pone.0004393

**Published:** 2009-02-06

**Authors:** Laurent Reber, Linda Vermeulen, Guy Haegeman, Nelly Frossard

**Affiliations:** 1 EA3771, Inflammation and Environment in Asthma, Université Louis Pasteur-Strasbourg-I, Faculté de Pharmacie, Illkirch, France; 2 LEGEST, Department of Molecular Biology, Ghent University, Ghent, Belgium; Northwestern University, United States of America

## Abstract

Transcription of the mast cell growth factor SCF (stem cell factor) is upregulated in inflammatory conditions, and this is dependent upon NF-κB, as well as the MAP kinases p38 and ERK activation. We show here that the MAPK downstream nuclear kinase MSK1 induces NF-κB p65 Ser276 phosphorylation upon IL-1ß treatment, which was inhibited in cells transfected with a MSK1 kinase-dead (KD) mutant compared to the WT control. In addition, we show by ChIP experiments that MSK1 as well as MAPK inhibition abolishes binding of p65, of its coactivator CBP, and of MSK1 itself to the κB intronic enhancer site of the SCF gene. We show that interaction between NF-κB and CBP is prevented in cells transfected by a p65 S276C mutant. Finally, we demonstrate that both transfections of MSK1-KD and MSK1 siRNA - but not the WT MSK1 or control siRNA - downregulate the expression of SCF induced by IL-1ß. Our study provides therefore a direct link between MSK1-mediated phosphorylation of Ser276 p65 of NF-κB, allowing its binding to the SCF intronic enhancer, and pathophysiological SCF expression in inflammation.

## Introduction

The nuclear factor-κB (NF-κB) family is composed of homodimers and heterodimers of the Rel family proteins, including p65 (RelA), c-Rel, RelB, p52 and p50 (for review, see [1]). The most abundant form of NF-κB is a heterodimer with two subunits: one p50 and one p65. NF-κB is bound to inhibitory IκB proteins in the cytoplasm. After stimulation by a variety of stimuli, NF-κB is released and translocates to the nucleus where it binds to its coactivators, mainly CBP (CREB-Binding Protein), and activates expression of pro-inflammatory genes, including the mast cell growth factor stem cell factor (SCF) [2].

NF-κB is activated by phosphorylation, which plays a key role in the regulation of its transcriptional activity, and is associated with nuclear translocation, CBP recruitment and DNA-binding activity (for review, see [3]). Phosphorylation of p65 occurs on several serine residues. For instance, upon treatment with TNFα, Ser529 is phosphorylated by casein kinase II [4], Ser536 by the IκB kinase (IKK) complex [5], Ser311 by protein kinase C (PKC)-ζ [6], and Ser276 by both PKA and mitogen- and stress-activated protein kinase 1 (MSK1) [7], [8].

MSK1 has a nuclear localization, and might therefore be an end-kinase in the inflammatory process involving NF-κB. We therefore focused our work on the MSK1-induced NF-κB activation as an approach of the potential role for MSK1 in inflammation. To do so, we used the SCF gene, to which p65 binds in cells stimulated by the pro-inflammatory cytokine IL-1ß. In this inflammation model, NF-κB activation totally controls SCF upregulated expression and the MAP kinases p38 and ERK, which are the direct activators of the nuclear kinase MSK1 [9], [10], also mediates this upregulation [2].

## Results

### Binding of the NF-κB complex to the κB site of the SCF gene

**Figure 1:**
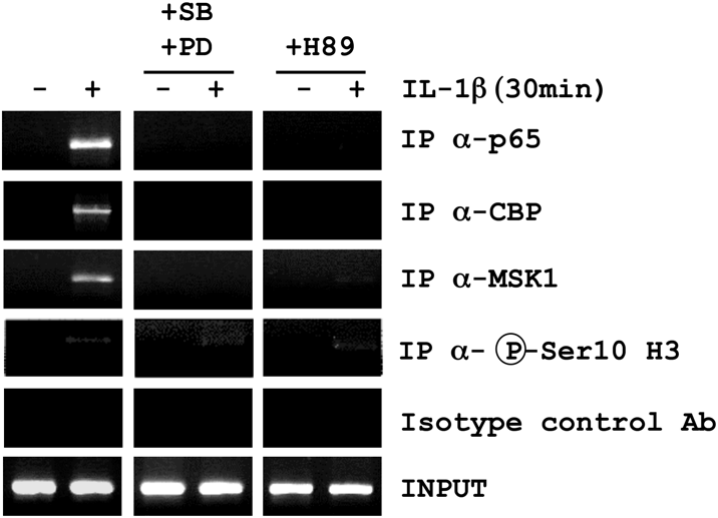
Effect of MAP kinase and MSK1 inhibitors on IL-1β-induced p65, MSK1 and CBP binding to the κB site of the SCF intronic enhancer. Human lung fibroblasts in culture were pre-incubated for 1 h with a combination of the p38 inhibitor SB202190 (SB; 3.5 µM) and the MEK inhibitor PD98059 (PD; 20 µM) or with the MSK1-PKA inhibitor H89 (10 µM) and treated with IL-1β (20 U/ml) for 30 min. The ChIP experiment was performed with anti-p65, MSK1, CBP, phospho-Ser10 histone H3 and control Ig antibodies. Co-immunoprecipitated genomic DNA fragments were amplified by PCR with SCF intronic enhancer-specific primers. Input reflects the relative amounts of sonicated DNA fragments before immunoprecipitation. Results are representative of 3 independent experiments performed in fibroblasts from 3 different donors.

We first show by ChIP experiments that p65 localizes to the κB intronic enhancer site of the SCF gene upon IL-1β treatment of human lung fibroblasts in primary culture (Figure 1). We further show the co-immunoprecipitation of p65, CBP, MSK1, and Ser10-phosphorylated histone H3 at this site. We further report that binding of p65, CBP and MSK1 is totally blocked by either inhibiting the MSK1 upstream kinases, p38 and ERK1/2 by use of their inhibitors SB202190 (3.5 µM) and PD98059 (20 µM), or by use of a non-selective MSK1-PKA inhibitor, compound H89 (10 µM). By contrast, phosphorylation of Ser10 histone H3 at the κB site of the SCF gene was unchanged (Figures 1 and S1). These results clearly suggest an interaction complex involving p65, CBP and MSK1 at this κB site dependent on MSK1 activity.

### Control of NF-κB activation by MSK1

**Figure 2:**
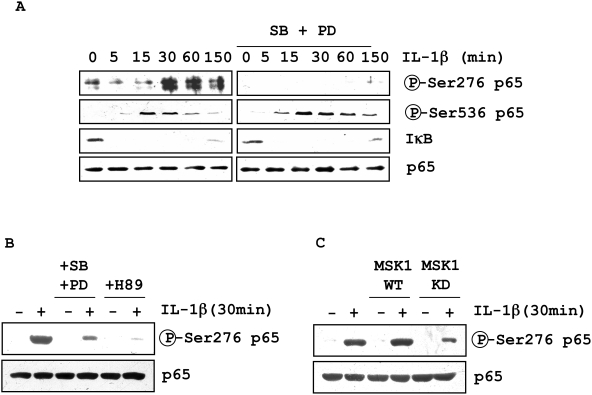
Effect of MAP kinase and MSK1 inhibition on IL-1β-induced NF-κB activation. *A.* Human lung fibroblasts in culture were pre-incubated for 1 h with a combination of SB202190 (SB; 3.5 µM) and PD98059 (PD; 20 µM) and treated with IL-1β for the indicated time. Western blot analysis used anti-phospho-Ser276-p65, anti-phospho-Ser536-p65, anti-IκB and anti-p65 antibodies. *B.* Cells were transfected with 1 µg of pHis-p65 plasmid (WT p65 subunit with a N-terminal His-Tag) and pre-incubated with a combination of SB and PD, or H89 (10 µM) for 1 h before IL-1ß treatment. *C.* Cells were co-transfected with His-p65 plasmid and WT or “kinase-dead” (KD) MSK1 plasmid (1 µM), and treated with IL-1β (20 U/ml). Results are representative of three independent experiments performed in fibroblasts from three different donors.

We used phospho-specific antibodies raised against phospho-Ser276- and phospho-Ser536-p65 to assess the involvement of MSK1 and its upstream kinases p38 and ERK on p65 phosphorylation in human lung fibroblasts in primary culture. IL-1β treatment led to p65 phosphorylation at both serine 536 and serine 276 in a time-dependent manner. Maximum phosphorylation was found after 15 and 30 min for Ser536 and Ser276, respectively (Figure 2A). Inhibitors of the upstream p38 and ERK MAPK, SB202190 and PD98059, totally abolished the IL-1β-induced p65 phosphorylation at Ser276 without any effect on Ser536 phosphorylation (Figure 2A). As a control, we also confirmed that these MAPK inhibitors had no effect on IκB degradation and subsequent NF-κB nuclear translocation (Figures 2A and S2). The non-selective inhibitor of MSK1-PKA, compound H89, also blocked the p65 Ser276 phosphorylation (86% inhibition, figure 2B). To confirm the involvement of MSK1, we used fibroblasts transfected with a kinase-dead mutant of MSK1 (MSK1 KD). In fibroblasts transfected with MSK1 KD, a 63% inhibition of the Ser276 phosphorylation induced by IL-1β was observed, whereas transfection with the MSK1 WT plasmid had no effect (Figure 2C). This clearly demonstrates that MSK1 controls NF-κB activity through phosphorylation of p65 Ser276.

### Control of p65 - CBP interaction by MSK1

**Figure 3:**
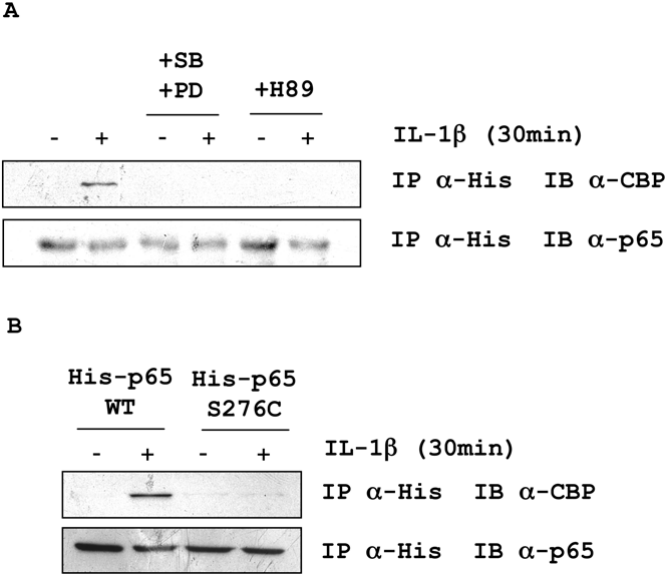
Effect of MSK1-mediated p65 Ser276 phosphorylation on IL-1β-induced p65-CBP interaction. *A.* Human lung fibroblasts in culture were transfected with 1 µg of pHis-p65 plasmid (WT p65 subunit with a N-terminal His-Tag), and pre-incubated for 1 h with a combination of SB202190 (SB; 3.5 µM) and PD98059 (PD; 20 µM), or with H89 (10 µM) and treated with IL-1β (20 U/ml). *B.* Human lung fibroblasts in culture were transfected with pHis-p65 or pHis-p65 S276C plasmids (1 µM), and treated with IL-1β (20 U/ml). His-p65 and co-precipitating CBP were revealed by immunoblotting using anti-p65 and anti-CBP antibodies. IP: immunoprecipitation; IB: immunoblot. Results are representative of three independent experiments performed in fibroblasts from three different donors.

Because inhibition of MSK1 impaired CBP recruitment at the SCF intronic enhancer (Figure 1), we investigated whether this kinase played a role in the interaction between NF-κB and CBP during IL-1β treatment. Human lung fibroblasts were transfected with a His-p65 plasmid. We found that CBP co-immunoprecipitated with the His-p65 subunit after IL-1β treatment (Figure 3A). This interaction was totally blocked when the cells were pre-incubated with either the MSK1-PKA inhibitor H89 or with inhibitors of its upstream kinases p38 and ERK, SB202190 and PD98059. To assess whether the p65 phosphorylation at Ser276 played a role in this interaction, p65-CBP co-immunoprecipitation was studied in fibroblasts transfected with a S276C His-p65 mutant or its control WT His-p65 plasmid and stimulated with IL-1ß. Mutation of serine 276 to cysteine totally abolished the p65-CBP co-immunoprecipitation (Figure 3B). This shows that phosphorylation of p65 Ser276 by MSK1 mediates NF-κB-CBP interaction in human lung fibroblasts.

### Control of SCF expression by MSK1

**Figure 4:**
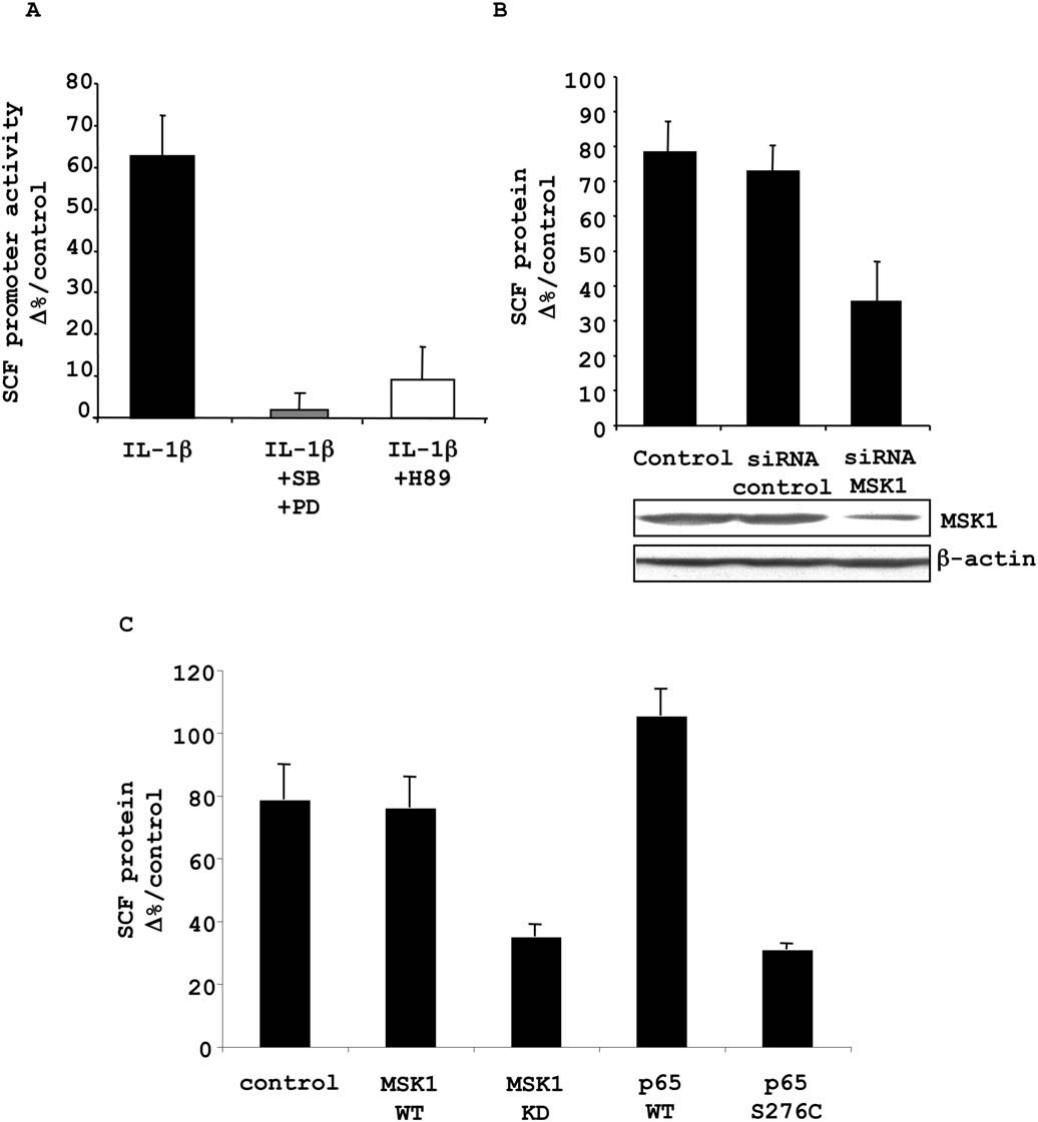
Effect of MSK1-mediated p65 Ser276 phosphorylation in IL-1β-induced SCF expression. *A.* Human lung fibroblasts in culture were transiently co-transfected with the pGL3e/SCF firefly luciferase construct and a *Renilla* luciferase construct (pRL-TK) as an internal control. Cells were pre-incubated for 1 h with a combination of SB202190 (SB; 3.5 µM) and PD98059 (PD; 20 µM) or with H89 (10 µM) and treated with IL-1β (20 U/ml). After 150 min, cells were harvested for luciferase activity measurement. The results are expressed as the level of pGL3e/SCF constructions' promoter-driven firefly luciferase expression after correcting for the transfection efficiency by pRL-TK luciferase measurements and represented as a percentage of control values. *B.* Fibroblasts were transfected with control and anti-MSK1 siRNA (100 nM), or transfection medium alone (control). After 48 hours, inhibition of MSK1 with siRNA was controlled by Western blotting in the cell lysate, using anti-MSK1, with anti-β-actin antibodies as a deposit control. Cells were treated with IL-1β (20 U/ml). SCF protein levels were assessed in the supernatant 5 hours after treatment by ELISA. *C*. Fibroblasts were transfected with WT or “kinase-dead” (KD) MSK1 plasmid (1 µg), WT or S276C p65 plasmids or transfection medium alone (control), and treated with IL-1β (20 U/ml). SCF protein levels were assessed by ELISA in the supernatant obtained 5 hours after treatment. Results are expressed as percentages of control values of three independent experiments performed in fibroblasts from three different donors.

We next investigated the physiological consequence of NF-κB activation by MSK1 on SCF expression. First, we assessed the effect of MSK1 on SCF promoter activity. Fibroblasts were transfected with a plasmid encoding the promoter region of the SCF gene, including exon 1 and the part of intron 1 containing the κB enhancer site, upstream from a luciferase reporter gene (pGL3e/SCF), as previously reported [2]. The MSK1-PKA inhibitor H89 or the MAPK inhibitors had no effect on the baseline luciferase reporter gene activity (Table S1). IL-1β increased the SCF promoter activity by 62% at 150 min, the time of maximum SCF mRNA expression previously reported [11], while H89 and the inhibitors of the upstream MAPK SB202190 and PD98059 totally abolished the IL-1β-induced luciferase reporter activity (figure 4A).

Next, we evaluated the involvement of MSK1 and p65 Ser276 phosphorylation on SCF protein expression. Since H89 is a non-selective inhibitor of MSK1, we added transfection strategies using either siRNA against MSK1, MSK1 Kinase-Dead (KD) or S276C p65 mutant plasmids, as compared to their respective controls. Baseline levels were not affected by any of the transfections (Table S2 and S3). Anti-MSK1 siRNA reduced MSK1 protein levels by 60% compared to the irrelevant control siRNA. It inhibited SCF expression by 51% compared with the control siRNA (Figure 4B).

In addition, transfection of fibroblasts with the MSK1 WT or KD plasmids led to a 7- and 8-fold MSK1 overexpression, respectively (Figure S3). MSK1 KD inhibited the SCF expression by 54% as compared to WT MSK1 or to control expression in untransfected cells (Figure 4C). Finally, S276C p65 transfection, inducing a 4-fold protein increase (Figure S3), inhibited SCF expression by 71% as compared to the p65 WT (figure 4C). Altogether, our results clearly show that MSK1 controls SCF protein expression through phosphorylation of p65 at Ser276.

## Discussion

We previously reported that the stem cell factor (SCF) gene activation is regulated through a functional κB enhancer site originally located in the first intron (+215-GGGAGCTCCC-+225) of the SCF gene [2]. The first important finding of the present study is that this κB intronic enhancer site is occupied by NF-κB and its coactivator CBP upon IL-1β-treatment of primary human lung fibroblasts, and that MSK1 also co-immunoprecipitates at this site. This suggests formation of a transcription complex involving MSK1 at the SCF κB enhancer site in inflammatory conditions. We additionally clearly show that this is dependent upon phosphorylation of the Ser276 residue of the p65 subunit of NF-κB, and that MSK1 is responsible for this phosphorylation. We demonstrate for the first time that this is a prerequisite for upregulated SCF expression in inflammation.

Another important finding of this paper is the suppression of binding of NF-κB, CBP and MSK1 to the SCF intronic enhancer after inhibition of the MAPKs p38 and ERK. These two MAPKs are reported to activate MSK1 in inflammatory conditions in cell lines like HEK293 and L929sA [7]. We additionally show that this colocalization is suppressed by the non-selective MSK1-PKA inhibitor H89, indicating that the in vivo occupancy of the SCF intronic enhancer by NF-κB and CBP is dependent upon MSK1 activation.

Transcriptional activity of NF-κB is controlled by phosphorylation of p65 at multiple serine residues. Here we show that p65 is phosphorylated at Ser276 and Ser536 upon IL-1β-treatment in human lung fibroblasts. However, inhibition of p38 and ERK MAP kinases had no effect on p65 Ser536 phosphorylation. This contrasts with p65 Ser536 phosphorylation by the p38 pathway in human bronchial epithelial BEAS-2B cells after pneumococci stimulation [12], or by the MEK-ERK pathway in rat vascular smooth muscle cells in primary culture after angiotensin II treatment [13]. We can deduce that different pathways may be involved in Ser536 phosphorylation, depending on the stimulus or cell type studied, which is a new and potently important information.

By contrast, we importantly report that inhibition of the MAPKs p38 and ERK or of the downstream kinase MSK1 blocks the phosphorylation of p65 Ser276 induced by IL-1β. This is consistent with direct p65 phosphorylation by MSK1 at Ser276 by IL-1β, as was also the case for TNFα, another inflammatory cytokine, in human HEK293 and murine L929sA cells [7]. This is also in accordance with the phosphorylation of the equivalent sites at p50 (Ser337) and c-Rel (Ser267) of the NF-κB protein family [14] during LPS treatment [15], [16], [17], [18], although phosphorylation was dependent on a PKAc subunit. Interestingly, PKAc also leads to p65 Ser276 phosphorylation in response to LPS in murine 70Z/3 pre-B cells [8], [19]. The possibility that PKAc might phosphorylate p65 at Ser276 cannot totally be ruled out by our studies, since H89 also inhibits PKA [20]. For this reason, we ascertained the role of MSK1 by transfection of a kinase-dead mutant of MSK1 (MSK1 KD), inducing inhibition of Ser276 phosphorylation in response to IL-1β, showing an important reduction (63%) taking into account the transfection efficiency (70%, data not shown) and the competition obtained (MSK1 KD *vs* endogenous MSK1).

Phosphorylation of p65 at Ser276 (as well as the analogous site in c-Rel) is reported to disrupt the interaction between its C-terminal and N-terminal regions, thus unmasking an interaction site with the histone acetyl transferase CBP [8], [15]. In the present study, co-immunoprecipitation experiments demonstrate that p65-CBP interaction is directly dependent upon prior p65 phosphorylation at Ser276 by MSK1. This result conforts the finding that acetylation of p65 at Lys310 by CBP is secondary to Ser276 phosphorylation [21]. This acetylation of p65 enhances its DNA-binding activity [22]. Similarly, acetylation of p50 and p52 by CBP is linked to DNA-binding at the iNOS and COX-2 promoter [23], [24], [25]. Thus we may postulate that NF-κB and CBP interaction with the SCF intronic enhancer is a direct consequence of Ser276 phosphorylation. This is parallel to reports that phosphorylation at Ser337 of the p50 subunit also controls DNA binding of p50 [16], [17]. Interestingly, the sequence surrounding p50 Ser337 (LRRKS^337^DLE) is highly similar to that around p65 Ser276 (LRRPS^276^DRE) [17]. Moreover, site-specific phosphorylation is reported to target NF-κB to a particular subset of genes [14]. Indeed, S276A mutation of p65 inhibits a reporter gene harboring an IL-6-derived κB site (GGGATTTTCC), while it has no effect when a palindromic κB site derived from the p52/p100 promoter (GGGAATTCCC) is concerned. We here report that the GGGAGCTCCC κB site of the SCF gene is also dependent upon Ser276 p65 phosphorylation, and that this determines NF-κB binding.

MSK1 also promotes phosphorylation of histone H3 at Ser10 [10], [26], depending on the gene, cell type and stimulus. We asked the question of the role of such MSK1-mediated chromatin modification in p65 DNA binding to the SCF gene, that would therefore affect the accessibility of the κB site. However, ChIP experiments show the absence of modification of H3 Ser10 phosphorylation when MSK1 or its upstream MAPK are inhibited. Since Duncan and collaborators also reported MSK1 not to phosphorylate histone H3 after TNFα treatment [27], we may submit that histone H3 phosphorylation is independent of the MSK1 pathway in inflammation. MSK1-mediated control of p65 binding to the SCF intronic enhancer is thus more likely to be due to Ser276 phosphorylation rather than chromatin remodelling.

Confirming this hypothesis, we finally demonstrate the direct involvement of MSK1-mediated p65 Ser276 phosphorylation in SCF expression. First, blocking the action of MSK1 by either a kinase-dead MSK1 mutant plasmid or anti-MSK1 siRNA leads to inhibition of IL-1ß-induced SCF expression. Second, SCF production is inhibited by mutation of the Ser276 to Cys. Both serve as the final demonstration that this Ser276 phosphorylation by MSK1 is a necessary requisite for SCF expression in inflammation.

**Figure 5:**
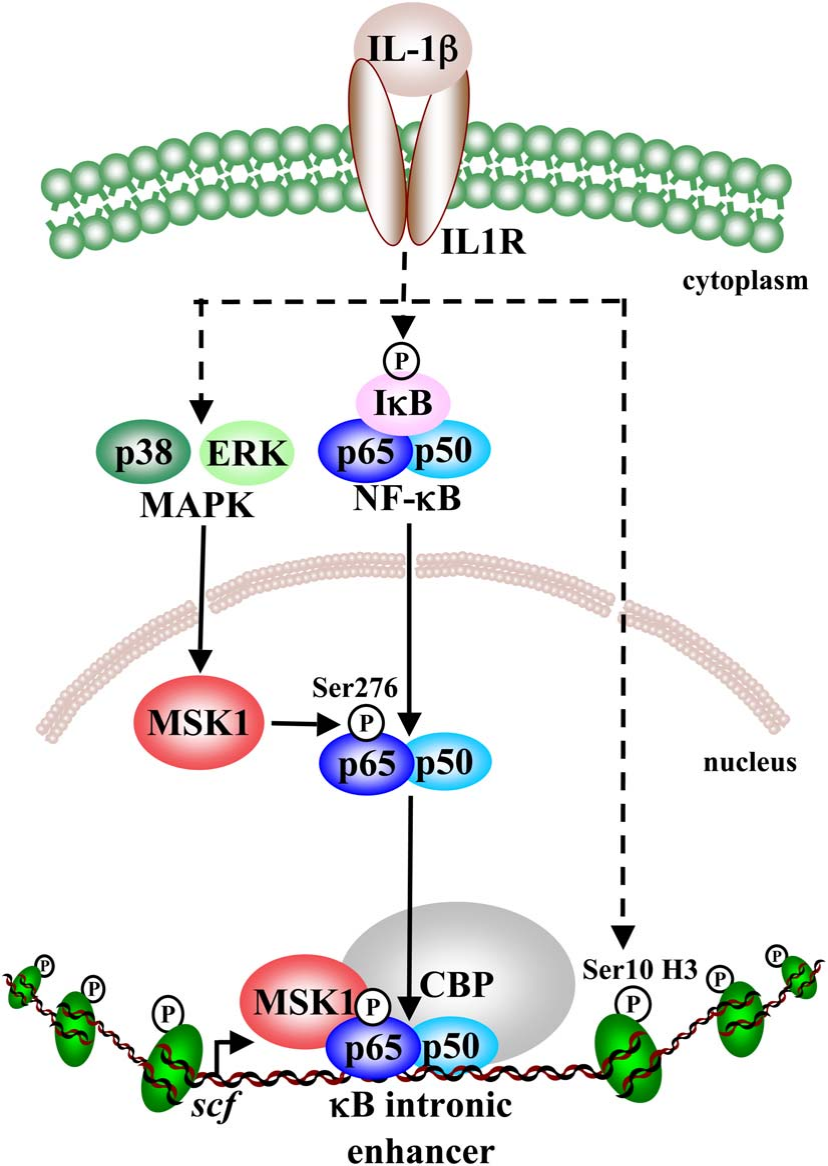
Pathway leading to SCF expression in primary human lung fibroblasts upon IL-1β treatment. After treatment with IL-1β, both p38 and ERK MAPK activate MSK1 which phosphorylates nuclear p65 at Ser276. This leads to binding of NF-κB p65 to the κB site of the SCF intronic enhancer, and to interaction with the coactivator CBP. Phosphorylation of histone H3 at Ser10 upon IL-1β-treatment is mediated through a MSK1-independent pathway. IL-1β: interleukin-1β; IL-1R: interleukin-1 receptor; ERK: extracellular signal regulated kinase; MAPK: mitogen-activated protein kinase; NF-κB: nuclear factor-κB; IκB: inhibitor of NF-κB; MSK1: mitogen- and stress-activated protein kinase 1; CBP: CREB binding protein; H3: histone 3.

In conclusion, we propose a model according to which MSK1 interacts with the enhanceosome and mediates both the binding of NF-κB to the SCF intronic enhancer and its interaction with its coactivator CBP (Figure 5). MSK1 may therefore be an interesting therapeutic target for inflammatory diseases, in view of its central role in NF-κB activation.

## Materials and Methods

### Culture of human primary lung fibroblasts

Human lung-derived fibroblasts were obtained by the explant technique, as described previously [11], [28]. Fibroblasts were cultured in DMEM/F-12 (1∶1) medium (Gibco BRL, Cergy Pontoise, France), supplemented with 10% fetal calf serum (FCS), penicillin (50 U/ml) and streptomycin (50 µg/ml), and incubated in a humidified mixture of 95% air and 5% CO_2_ at 37°C. They were subsequently split 1∶4 at confluence and passaged. Fibroblasts were used at passage 7.

### Cell treatment

At confluence, fibroblasts were starved for 48 h. MAP kinase pathways were studied with the p38 inhibitor SB202190 (3.5 µM) and the MEK inhibitor PD98059 (20 µM) (both from Calbiochem, La Jolla, CA). MSK1 was inhibited with the compound H89 (10 µM) (Sigma Chemicals, St. Louis, MO). All these molecules were incubated for 1 h before IL-1β treatment. Human recombinant IL-1β (Roche Diagnostics) was added at a final concentration of 20 U/ml. Control treatments used solvent, DMSO (0.1%), and had no effect on SCF promoter activity, p65 phosphorylation or SCF expression.

### Plasmids

The pGL3e/SCF reporter plasmid was described previously [2]. pHis-p65 (full-length p65 subunit fused in phase to the N-terminal histidine Tag), pMSK1-E (named MSK1 WT in this paper, and coding for human MSK1 C-terminally fused to an E-Tag), pMSK1-E-D565A (kinase-dead mutant of MSK1, named MSK1 KD in this paper), pRcRSVp65 (named p65 WT in this paper, coding for the p65 subunit under the control of the RSV-LTR promoter) and pRcRSVp65S276C (named p65 S276C in this paper) were all deposited by Dr. Linda Vermeulen and Prof. Guy Haegeman at the Belgian Coordinated Collections of Micro-organisms BCCM plasmid bank. His-tagged forms of the latter two plasmids (His-p65 WT and His-p65S276C in this paper) were constructed by exchanging the HindIII-Eco91I fragent of the pHis-p65.

### Cell transfection

Transient transfection of primary lung fibroblasts was performed with the Nucleofector® kit (Amaxa AG, Germany). Plasmid DNA (1 µg), control siRNA (100 nM) (sc-37007, Santa Cruz Biotechnology, Santa Cruz, CA) or MSK1 siRNA (100 nM) (sc-35977, Santa Cruz Biotechnology) was added to 1.10^6^ cells suspended in 100 µl of Basic Nucleofector® solution for primary mammalian fibroblasts. The U-23 program (Amaxa AG, Germany) was selected for optimal density of transfection (70%) and cell survival (68%), according to the manufacturer's instructions. Cells were then plated in 6-well plates and serum-starved for 48 h before experiments, with medium changed 24 h after transfection. The green fluorescent protein (GFP) plasmid provided in the Nucleofector® kit was used to determine the transfection efficiency, according to the manufacturer's instructions.

### Preparation of cytoplasmic and nuclear extracts

Fibroblasts were rinsed in cold PBS and lysed in a solution containing 0.6% Nonidet P-40, 10 mM KCl, 10 mM HEPES, 0.1 mM EDTA (all products from Sigma Aldrich), and Complete™ Mini-EDTA-free protease inhibitor cocktail (Roche Diagnostics). After centrifugation (30 s, 2000 *g*), supernatants were incubated on ice for 5 min. Nuclei were precipitated by centrifugation (4°C, 3 min, 15000 *g*), supernatants collected as cytosolic extracts, and the nuclei resuspended in a solution of 10% glycerol, 20 mM HEPES, 400 mM NaCl, 1 mM EDTA and Complete™ Mini-EDTA-free protease inhibitor cocktail. The mixture was incubated on ice for one hour, the supernatant collected after centrifugation for 5 min at 15000 *g*, and saved as nuclear extracts.

### Immunoprecipitation

Cells were rinsed in cold PBS and lysed in a RIPA buffer (see § Western Blotting) or in co-immunoprecipitation buffer (for study of p65/CBP interaction) containing 200 mM NaCl, 20 mM Tris-HCl (pH 7.5), 1% Triton X-100, 1 mM DTT and Complete™ Mini-EDTA-free protease inhibitor cocktail (1 tablet per 10 ml) (Roche Diagnostics, all other products from Sigma Aldrich). Cell lysate was kept on ice for 10 min and centrifuged for 10 min at 14 000 *g*, 4°C. 1 µg of anti-His-Tag antibody (Invitrogen) was added to 200 µl of cell lysate and incubated overnight at 4°C. 20 µl of protein A agarose beads (50% bead slurry) was added for 3 hours at 4°C. Samples were centrifuged for 30 seconds at 4°C. Pellets were washed five times with lysis buffer and resuspended in RIPA buffer. Laemmli buffer was added and samples boiled at 100°C for 5 min. Samples were centrifuged for 1 min at 14 000 *g*, and supernatants analysed by Western blotting.

### Western Blotting

The fibroblasts were rinsed in cold PBS and lysed in a RIPA buffer containing 50 mM Tris–HCl pH 7.4, 150 mM NaCl, 1 mM EDTA, 1 mM PMSF, 1% Nonidet P-40, 0.25% sodium deoxycholate, 1 mM NaF, 1 mM Na_3_VO_4_ (all other products from Sigma Aldrich), and Complete™ Mini-EDTA-free protease inhibitor cocktail (Roche Diagnostics). After centrifugation (30 min, 12 000 *g*, +4°C), the protein concentration was measured by the BCA reagents (Sigma Aldrich). Total protein (50 µg) was subjected to 8-16% SDS-PAGE gel and then blotted onto nitrocellulose. Immunoblotting used the following antibodies: rabbit anti-human IκB-α polyclonal antibody (1/1000, Calbiochem, La Jolla, CA), mouse anti-human phospho- IκB-α monoclonal antibody, (1/1000, Ab-1, Oncogene Research Product, Boston, MA), rabbit anti-human phospho-Ser276 p65 antibody (1/1000, 3037, Cell Signaling Technology, Danvers MA), rabbit anti-human phospho-Ser536 p65 antibody (1/1000, 3031, Cell Signaling Technology), rabbit anti-human p65 polyclonal antibody (1/200, sc-109, Santa Cruz Biotechnology, Santa Cruz, CA), rabbit anti-human CBP polyclonal antibody (1/200, sc-369, Santa Cruz Biotechnology), mouse anti-human β-actin monoclonal antibody (1/5000, Ab-1, Oncogene Research Product), goat anti-human MSK1 (1/200, sc-9392, Santa Cruz Biotechnology. Immunoreactive proteins were detected with ECL™ reagents (Amersham Biosciences, Orsay, France).

### Analysis of reporter gene and SCF expression

Reporter gene assays were carried out as described previously [2]. SCF production was measured with an ELISA procedure. It used a capture anti-human SCF monoclonal antibody (clone 13302.6; R&D Systems Europe, Abingdon, UK) and a biotinylated detection anti-human SCF polyclonal antibody (R&D Systems Europe), revealed by extravidin-horseradish peroxidase and a 3,3′,5,5′-tetramethylbenzidine liquid substrate system (Sigma Chemicals). Standard curves were generated with recombinant human SCF (R&D Systems Europe) diluted in the starving medium and were linear from 3.9 to 500 pg/ml.

### Chromatin immunoprecipitation

ChIP assays were performed with a previously described protocol (Upstate Biotechnology) with modifications. Following treatments, 1.10^7^ adherent fibroblasts per condition were washed with ice-cold PBS and the cross-linking buffer (100 mM NaCl, 0.5 mM EGTA, 1 mM EDTA, 50 mM Hepes, pH 9). DNA-protein interactions were then cross-linked for 20 min at room temperature by the addition of formaldehyde, to a final concentration of 1%. Cross-linking was stopped by addition of glycine to a final concentration of 125 mM for 5 min. The cell monolayers were rinsed twice with ice-cold PBS, scraped into conical tubes, and centrifuged at 1500 rpm for 5 min at room temperature. Pellets were aspirated and resuspended in SDS lysis buffer (1% SDS, 10 mM EDTA, 50 mM Tris, pH 8.1), and incubated for 10 min on ice. Samples were sonicated (4 times, 20 s each, 60%, Vibracell, Bioblock Scientific). This procedure resulted in DNA fragment sizes of 0.2–1 kb. Samples were then centrifuged (16 000 *g*, 10 min, 4°C) into a swinging rotor. Supernatants were nutated for 1 h at 4°C with 50 ml of 50% slurry Protein G Agarose/Salmon sperm DNA in SDS lysis buffer (Upstate, Lake Placid, NY). After centrifugation at 1500 rpm for 5 min, 1 mg of anti-p65, anti-MSK1, anti-CBP, anti-phospho-Ser10 H3 or isotype control antibodies was added to 500 µl aliquots of pre-cleared chromatin extract and incubated and rotated at 4°C overnight. 60 ml of protein G agarose/Salmon sperm DNA was added and the samples incubated and rotated for 1 h at 4°C. After centrifugation (1000 rpm, 4°C, 1 min), pellets were washed for 5 min with Low Salt immune Complex Wash Buffer (0.1% SDS, 1% Triton X-100, 2 mM EDTA, 20 mM Tris-HCl, pH 8.1, 150 mM NaCl), High Salt Immune Complex Wash Buffer (0.1% SDS, 1% Triton X-100, 2 mM EDTA, 20 mM Tris-HCl, pH 8.1, 500 mM NaCl), LiCl Immune Complex Wash Buffer (0.25 mM LiCl, 1% IGEPAL-CA630, 1% deoxycholic acid, 1 mM EDTA, 10 mM Tris, pH 8.1) and twice with Tris-EDTA Buffer (10 mM Tris-HCl, 1 mM EDTA, pH 8.0). Histone complexes were eluted by adding 500 µl of Elution Buffer (1% SDS, 0.1 M NaHCO_3_). After centrifugation, 20 µl of 5 M NaCl was added to the supernatant and histone-DNA crosslinks reversed by heating at 65°C overnight. 10 µl of 0.5 M EDTA, 20 µl of 1 M Tris-HCl, pH 7.5 and 2 µl of 10 mg/ml Proteinase K were added and the samples placed at 55°C for 3 h. DNA was phenol-CHCl_3_-isoamylalcohol (50∶49∶1)-extracted once, CHCl_3_-extracted once and ethanol-precipitated. Pellets were resuspended in 20 µl of sterile water. PCR reactions (50 µl) were programmed for 35 cycles with 2 µl of DNA sample and 0.2 mM of each appropriate primer oligonucleotide. Titrations were performed to ensure a linear range of amplification. The κB site located in the first intron of the SCF gene (+215/+225) was amplified with the PCR primer pairs 5′-CTTGCCTGCTTCTCGCCTACCC-3′ and 5′-CCCCGCATCCTCTTGTCTCACC-3′ (position −111 and +315).

## Supporting Information

Figure S1 Effect of MAP kinase and MSK1 inhibitors on IL-1β-induced histone H3 phosphorylation. Human lung fibroblasts in culture were pre-incubated for 1 h with a combination of SB202190 (SB; 3.5 µM) and PD98059 (PD; 20 µM) or with H89 (10 µM) and treated with IL-1β for 30 min. The cell lysates underwent 16% SDS-PAGE electrophoresis and were transferred onto a nitrocellulose membrane. Western blot analysis used anti-phospho Ser10 histone H3 antibodies and, as controls, anti-β-actin antibodies. Results are representative of three independent experiments performed in fibroblasts from three different donors.
(0.23 MB DOC)

Figure S2 Effect of MAPK inhibitors on IκB phosphorylation and NF-κB nuclear translocation. A. Fibroblasts were pre-treated for 1 h with the proteasome inhibitor MG132 (MG; 10 µM) alone (control) or in combination with SB202190 and/or PD98059. Cells were incubated with IL-1µ (20 U/ml) for 0–60 min. Western blot analysis used anti-phospho IκB antibodies, as well as anti-IκB antibodies as controls. B. Cells were pre-incubated for 1 h with SB202190, PD98059 or a combination of both and treated with IL-1µ for 0–30 min. The nuclear and cytoplasmic extracts underwent 10% SDS-PAGE electrophoresis and were transferred onto a nitrocellulose membrane. Western blot analysis used anti-p65, anti µ-actin and anti-CBP antibodies. Results are representative of three independent experiments performed in fibroblasts from three different donors.
(1.05 MB DOC)

Figure S3 Overexpression of WT or KD MSK1 and WT or S276C p65 plasmids. MSK1 and p65 protein levels were analysed 48h after transfection of lung fibroblasts by western blot. WT or KD MSK1 plasmids transfection led to MSK1 overexpression (7- and 8-fold, respectively). WT or S276C p65 plasmids transfection led both to a 4-fold overexpression of p65. Representative blot and ratio to µ-actin are shown.
(0.58 MB DOC)

Table S1 Activity of the pGL3e/SCF plasmid in the presence of the MAP kinase inhibitors SB202190 and PD98059 or the non selective MSK1/PKA inhibitor H89. Fibroblasts were co-transfected with the pGL3e/SCF firefly luciferase construct and a Renilla luciferase construct (pRL-TK) as an internal control. Forty-eight hours after transfection, cells were pre-incubated for 1 h with a combination of SB202190 (SB; 3.5 µM) and PD98059 (PD; 20 µM) or with H89 (10 µM) before treatment with IL-1β (20 U/ml). After 150 min, cells were harvested for luciferase activity measurement. The results are expressed as the level of pGL3e/SCF constructions' promoter-driven firefly luciferase expression after correcting for the transfection efficiency by pRL-TK luciferase measurements and represented as a percentage of control values. Results are means (blocks)±SE mean (bars) of three independent experiments performed in fibroblasts from three different donors.
(0.03 MB DOC)

Table S2 SCF levels in fibroblasts transfected with control or anti-MSK1 siRNA. Fibroblasts were transfected with control or anti-MSK1 siRNA (100 nM) transfection medium alone (“untransfected”). Forty-eight hours after transfection, cells were treated with IL-1β (20 U/ml). SCF protein levels (pg/ml) were assessed by ELISA in the supernatant obtained 5 hours after treatment. Results are means (blocks)±SE mean (bars) of three independent experiments performed in fibroblasts from three different donors.
(0.03 MB DOC)

Table S3 SCF levels in fibroblasts transfected with KD MSK1 or S276C p65 plasmids and their respective controls. Fibroblasts were transfected with “kinase-dead” (KD) or WT MSK1 plasmids (1 µg), S276C or WT p65 plasmids (1 µg) or transfection medium alone (“untransfected”). Forty-eight hours after transfection, cells were treated with IL-1β (20 U/ml). SCF protein levels (pg/ml) were assessed by ELISA in the supernatant obtained 5 hours after treatment. Results are means (blocks)±SE mean (bars) of three independent experiments performed in fibroblasts from three different donors.
(0.03 MB DOC)
