# Supplementary material for: Ser276 Phosphorylation of NF-kB p65 by MSK1 Controls SCF Expression in Inflammation
Source: PLoS One. 2009 Feb 6;4(2):e4393. doi: 10.1371/journal.pone.0004393 (PMC2632887; doi:10.1371/journal.pone.0004393)
Supplement: Figure S1 — Effect of MAP kinase and MSK1 inhibitors on IL-1β-induced histone H3 phosphorylation. Human lung fibroblasts in culture were pre-incubated for 1 h with a combination of SB202190 (SB; 3.5 µM) and PD98059 (PD; 20 µM) or with H89 (10 µM) and treated with IL-1β for 30 min. The cell lysates underwent 16% SDS-PAGE electrophoresis and were transferred onto a nitrocellulose membrane. Western blot analysis used anti-phospho Ser10 histone H3 antibodies and, as controls, anti-β-actin antibodies. Results are representative of three independent experiments performed in fibroblasts from three different donors. (0.23 MB DOC) [file pone.0004393.s001.doc]

**Figure S1**
