# Supplementary material for: Ser276 Phosphorylation of NF-kB p65 by MSK1 Controls SCF Expression in Inflammation
Source: PLoS One. 2009 Feb 6;4(2):e4393. doi: 10.1371/journal.pone.0004393 (PMC2632887; doi:10.1371/journal.pone.0004393)
Supplement: Figure S2 — Effect of MAPK inhibitors on IκB phosphorylation and NF-κB nuclear translocation. A. Fibroblasts were pre-treated for 1 h with the proteasome inhibitor MG132 (MG; 10 µM) alone (control) or in combination with SB202190 and/or PD98059. Cells were incubated with IL-1µ (20 U/ml) for 0–60 min. Western blot analysis used anti-phospho IκB antibodies, as well as anti-IκB antibodies as controls. B. Cells were pre-incubated for 1 h with SB202190, PD98059 or a combination of both and treated with IL-1µ for 0–30 min. The nuclear and cytoplasmic extracts underwent 10% SDS-PAGE electrophoresis and were transferred onto a nitrocellulose membrane. Western blot analysis used anti-p65, anti µ-actin and anti-CBP antibodies. Results are representative of three independent experiments performed in fibroblasts from three different donors. (1.05 MB DOC) [file pone.0004393.s002.doc]

**Figure S2**
