# Supplementary material for: Ser276 Phosphorylation of NF-kB p65 by MSK1 Controls SCF Expression in Inflammation
Source: PLoS One. 2009 Feb 6;4(2):e4393. doi: 10.1371/journal.pone.0004393 (PMC2632887; doi:10.1371/journal.pone.0004393)
Supplement: Figure S3 — Overexpression of WT or KD MSK1 and WT or S276C p65 plasmids. MSK1 and p65 protein levels were analysed 48h after transfection of lung fibroblasts by western blot. WT or KD MSK1 plasmids transfection led to MSK1 overexpression (7- and 8-fold, respectively). WT or S276C p65 plasmids transfection led both to a 4-fold overexpression of p65. Representative blot and ratio to µ-actin are shown. (0.58 MB DOC) [file pone.0004393.s003.doc]

**Figure S3**
