# Supplementary material for: Ser276 Phosphorylation of NF-kB p65 by MSK1 Controls SCF Expression in Inflammation
Source: PLoS One. 2009 Feb 6;4(2):e4393. doi: 10.1371/journal.pone.0004393 (PMC2632887; doi:10.1371/journal.pone.0004393)
Supplement: Table S1 — Activity of the pGL3e/SCF plasmid in the presence of the MAP kinase inhibitors SB202190 and PD98059 or the non selective MSK1/PKA inhibitor H89. Fibroblasts were co-transfected with the pGL3e/SCF firefly luciferase construct and a Renilla luciferase construct (pRL-TK) as an internal control. Forty-eight hours after transfection, cells were pre-incubated for 1 h with a combination of SB202190 (SB; 3.5 µM) and PD98059 (PD; 20 µM) or with H89 (10 µM) before treatment with IL-1β (20 U/ml). After 150 min, cells were harvested for luciferase activity measurement. The results are expressed as the level of pGL3e/SCF constructions' promoter-driven firefly luciferase expression after correcting for the transfection efficiency by pRL-TK luciferase measurements and represented as a percentage of control values. Results are means (blocks)±SE mean (bars) of three independent experiments performed in fibroblasts from three different donors. (0.03 MB DOC) [file pone.0004393.s004.doc]

**Table S1**

|  | **control** | **IL-1β** |
| --- | --- | --- |
| **Solvent** | 8.3±0.6 | 13.5±0.8 |
| **SB202190 + PD98059** | 8.4±0.4 | 8.7±0.6 |
| **H89** | 8.8±0.6 | 9.6±0.7 |
