# Supplementary material for: Ser276 Phosphorylation of NF-kB p65 by MSK1 Controls SCF Expression in Inflammation
Source: PLoS One. 2009 Feb 6;4(2):e4393. doi: 10.1371/journal.pone.0004393 (PMC2632887; doi:10.1371/journal.pone.0004393)
Supplement: Table S2 — SCF levels in fibroblasts transfected with control or anti-MSK1 siRNA. Fibroblasts were transfected with control or anti-MSK1 siRNA (100 nM) transfection medium alone (“untransfected”). Forty-eight hours after transfection, cells were treated with IL-1β (20 U/ml). SCF protein levels (pg/ml) were assessed by ELISA in the supernatant obtained 5 hours after treatment. Results are means (blocks)±SE mean (bars) of three independent experiments performed in fibroblasts from three different donors. (0.03 MB DOC) [file pone.0004393.s005.doc]

**Table S2**

|  | **control** | **IL-1β** |
| --- | --- | --- |
| **untransfected** | 15.2±0.3 | 27.1±1.3 |
| **control siRNA** | 17.0±1.3 | 29.4±1.2 |
| **anti-MSK1 siRNA** | 15.2±1.0 | 20.6±1.7 |
