# Supplementary material for: Ser276 Phosphorylation of NF-kB p65 by MSK1 Controls SCF Expression in Inflammation
Source: PLoS One. 2009 Feb 6;4(2):e4393. doi: 10.1371/journal.pone.0004393 (PMC2632887; doi:10.1371/journal.pone.0004393)
Supplement: Table S3 — SCF levels in fibroblasts transfected with KD MSK1 or S276C p65 plasmids and their respective controls. Fibroblasts were transfected with “kinase-dead” (KD) or WT MSK1 plasmids (1 µg), S276C or WT p65 plasmids (1 µg) or transfection medium alone (“untransfected”). Forty-eight hours after transfection, cells were treated with IL-1β (20 U/ml). SCF protein levels (pg/ml) were assessed by ELISA in the supernatant obtained 5 hours after treatment. Results are means (blocks)±SE mean (bars) of three independent experiments performed in fibroblasts from three different donors. (0.03 MB DOC) [file pone.0004393.s006.doc]

**Table S3**

|  | **control** | **IL-1β** |
| --- | --- | --- |
| **untransfected** | 16.7±0.6 | 29.8±1.9 |
| **WT MSK1** | 18.4±1.6 | 32.4±1.8 |
| **KD MSK1** | 15.2±0.5 | 20.5±0.6 |
| **WT p65** | 17.2±0.8 | 35.3±1.5 |
| **S276C p65** | 15.0±0.1 | 19.6±0.3 |
